# Supplementary material for: Performance of Coronary Angiography in the Detection of Coronary Artery Disease in Patients with Systolic Left Ventricular Dysfunction and No Prior Ischemic Heart Disease
Source: J Clin Med. 2022 Feb 18;11(4):1097. doi: 10.3390/jcm11041097 (PMC8880097; doi:10.3390/jcm11041097)
Supplement: Supplementary file 1 [file jcm-11-01097-s001.zip › jcm-1550734-supplementary.pdf]

**Table S1**. Significant Coronary Stenosis in patients with Heart Failure (SCS-HF) score.

| SCS-HF Score | |
|---|---|
| **Variable** | **Points** |
| Age ≤ 50 years | −2.5 |
| Atrial fibrillation/flutter | −3 |
| Female sex | −2.5 |
| Hypercholesterolemia | +2 |
| Diabetes mellitus | +2.5 |
| Segmentary alterations | +2.5 |
| Presence of Q wave | +5 |
| **Range of points** | −8 to 12 |

**Table S2.** Number of patients in Significant Coronary Stenosis in patients with Heart Failure (SCS-HF) score by non-significant and significant coronary stenosis.

| SCS-HF Score | Non-Significant Coronary Stenosis (N = 401) | Significant Coronary Stenosis (N = 135) | Total (N = 536) |
|---|---|---|---|
| **−8** | 1 | 0 | 1 |
| **−6** | 0 | 0 | 0 |
| **−4** | 14 | 0 | 14 |
| **−2** | 72 | 2 | 74 |
| **0** | 140 | 21 | 161 |
| **2** | 66 | 26 | 92 |
| **4** | 44 | 19 | 63 |
| **6** | 49 | 30 | 79 |
| **8** | 12 | 22 | 34 |
| **10** | 3 | 10 | 13 |
| **12** | 0 | 5 | 5 |
